# DeepGene: an advanced cancer type classifier based on deep learning and somatic point mutations

**DOI:** 10.1186/s12859-016-1334-9

**Published:** 2016-12-23

**Authors:** Yuchen Yuan, Yi Shi, Changyang Li, Jinman Kim, Weidong Cai, Zeguang Han, David Dagan Feng

**Affiliations:** 1 School of Information Technologies, The University of Sydney, Darlington, NSW 2008 Australia; 2 Key Laboratory of Systems Biomedicine, Shanghai Center for Systems Biomedicine, Shanghai Jiaotong University, Shanghai, 200240 China

## Abstract

**Background:**

With the developments of DNA sequencing technology, large amounts of sequencing data have become available in recent years and provide unprecedented opportunities for advanced association studies between somatic point mutations and cancer types/subtypes, which may contribute to more accurate somatic point mutation based cancer classification (SMCC). However in existing SMCC methods, issues like high data sparsity, small volume of sample size, and the application of simple linear classifiers, are major obstacles in improving the classification performance.

**Results:**

To address the obstacles in existing SMCC studies, we propose DeepGene, an advanced deep neural network (DNN) based classifier, that consists of three steps: firstly, the clustered gene filtering (CGF) concentrates the gene data by mutation occurrence frequency, filtering out the majority of irrelevant genes; secondly, the indexed sparsity reduction (ISR) converts the gene data into indexes of its non-zero elements, thereby significantly suppressing the impact of data sparsity; finally, the data after CGF and ISR is fed into a DNN classifier, which extracts high-level features for accurate classification. Experimental results on our curated TCGA-DeepGene dataset, which is a reformulated subset of the TCGA dataset containing 12 selected types of cancer, show that CGF, ISR and DNN all contribute in improving the overall classification performance. We further compare DeepGene with three widely adopted classifiers and demonstrate that DeepGene has at least 24% performance improvement in terms of testing accuracy.

**Conclusions:**

Based on deep learning and somatic point mutation data, we devise DeepGene, an advanced cancer type classifier, which addresses the obstacles in existing SMCC studies. Experiments indicate that DeepGene outperforms three widely adopted existing classifiers, which is mainly attributed to its deep learning module that is able to extract the high level features between combinatorial somatic point mutations and cancer types.

## Background

Cancer is known as a category of disease causing abnormal cell growths or tumors that potentially invade or metastasize to other parts of human body [1]. It has long become one of the major lethal diseases which leads to about 8.2 million, or 14.6%, of all human deaths each year [2].

To alleviate the impact of cancer to human health, considerable research endeavors have been devoted to the related diagnosis and therapy techniques, among which somatic point mutation based cancer classification (SMCC) is an important perspective. The purpose of SMCC is to detect the cancer types or subtypes based on somatic gene mutations from the patient, so that the cancer condition of the patient can be specified. Due to the drop in the cost of DNA sequencing in recent years, the availability of DNA sequencing data has increased dramatically, which greatly promotes the developments of SMCC [3]. Compared with conventional cancer classification methods that are mostly based on morphological appearances or gene expressions of the tumor, SMCC is particularly effective in differentiating tumors with similar histopathological appearances [4] and is significantly more robust to environmental influences, thus is favorable in delivering more accurate classification results. Other genetic aberrations such as copy number variance, translocation, and small insertion or deletion have also been shown to be associated with different cancers [5, 6], but due to the major causal role of somatic point mutations and potential application consideration, we only focus on this kind of genetic aberration in this study. Moreover, the combinatorial point mutation patterns learned in predicting cancer types/subtypes can be used for developing diagnostic gene marker panels that are cost effective. This is particularly true , when compared to DNA amplifications and rearrangements which usually require whole genome sequencing and is expensive for patients, especially regarding time series and whole genome sequencing used in tracing tumor linage evolution during cancer progression.

Clinically, SMCC may significantly facilitate cancer-related diagnoses and treatments, such as personalized tumor medicine [7], targeted tumor therapy [8] and compound medicine [9]. It can also aid cancer early diagnosis (CED) in combination with the sampling and sequencing of circulating tumor cells (CTCs) or circulating DNA (ctDNA) [10–12]. Given the promising applications above, SMCC is widely studied in recent researches [13–15].

In recent years, the drastic developments of machine learning methods have greatly facilitated the researches in bioinformatics, including SMCC. In order to predict the cancer types/subtypes more effectively, many machine learning approaches have been applied in existing cancer type prediction works, which have shown promising results [16–18]. Currently, remarkable developments have been demonstrated in tumor cases of colorectal [19], breast [20], ovary [21], brain [22], and melanoma [23]. However, there are at least three major unresolved challenges:

1. Normal sequencing results involve extremely large number of genes, usually in tens of thousands, but only a small discriminatory subset of genes is related to the cancer classification task. The other genes are largely irrelevant genes whose existence will only obstruct the cancer classification. Many recent works have been conducted in identifying the discriminatory subset of genes. For example, Cho et al. [24] apply the mean and standard deviation of the distances from each sample to the class center as criteria for classification; Yang et al. [25] improve the method in [24] and bring inter-class variations into the algorithm; Cai et al. [26] propose the clustered gene selection, which groups the genes via *k*-means clustering and picks up the top genes in each group that are closest to the centroid locations. These methods are simple and effective in some cases, but their heuristics are designed for continuous gene expression data, and are not directly applicable to discrete, and especially binary point mutation data.
2. Even within the discriminatory subset, the majority of genes are not guaranteed to contain informative point mutations and often remain normal (i.e. zero values in the data) [27], which results in extremely sparse gene data (even all-zeros) that is difficult to classify. Yet, to the best of our knowledge, there has been no existing work specifically devised for reducing the data sparsity for SMCC.
3. Different genes related to specific types of cancer are generally correlated and have complex interactions which may impede the application of conventional simple linear classifiers such as linear kernel support vector machine (SVM) [28]. Therefore, an advanced classifier being capable of extracting the high level features within the discriminatory subset is desired. Although there have been recent works utilizing sparse-coding [29] or auto-encoder [17] for gene annotation, no work has been devoted in applying high-level machine learning approaches to SMCC.

In recent years, the developments of deep neural network (DNN) [30] have equipped bioinformaticians with powerful machine learning tools. DNN is a type of artificial neural network that aims to model abstracted high-level data features using multiple nonlinear and complex processing layers, and provides feedback via back-propagation [31]. First introduced in 1989 [32], DNN has garnered tremendous developments and is widely applied in image classification [33, 34], object localization [35, 36], facial recognition [37, 38], etc. DNN has the potential to introduce novel opportunities for SMCC where it perfectly fits the need for large scale data processing and high level feature extraction. However, to the present, applying customized DNN on SMCC is yet to be explored.

In this paper, we propose a novel SMCC method, named DeepGene, designed to simultaneously address the three identified issues. DeepGene is a DNN-based classification model composed of three steps. It first conducts two pre-processing techniques, including the clustered gene filtering (CGF) based on mutation occurrence frequency, and the indexed sparsity reduction (ISR) based on indexes of non-zero elements; the gene data is then classified by a fully-connected DNN classifier into a specific cancer type. The proposed DeepGene model has four distinct contributions:

1. The proposed CGF procedure locates the discriminatory gene subset based on mutation occurrence frequency. CGF utilizes features from the whole dataset instead of the current sample alone (e.g. mean and standard deviation), and thus more objectively reflects the correlations among the genes which can more effectively summarize the discriminatory subset. In addition, CGF does not require any prior knowledge from the original data and therefore functions well on both discrete and binary point mutation data.
2. The proposed ISR procedure converts the sparse gene data into indexes of its non-zero elements. ISR eliminates the vast majority of zero gene elements, and significantly reduces the complexity of the gene data during such process.
3. We establish a fully connected DNN classifier that uses the gene data after CGF and ISR for cancer classification. With the capacity of high-level feature extraction, our classifier is able to effectively extract deep features from the complexly correlated gene data, and significantly improve the classification accuracy compared with conventional simple linear classifiers such as SVM.
4. We compile and release the TCGA-DeepGene dataset, which is a reformulated subset of the widely applied TCGA dataset [39] in genome-related researches. TCGA-DeepGene selects 22,834 genes of 12 types of cancer from 3122 different samples, and regularizes the data in a unified format so that classification tasks can be readily performed.

**Fig. 1:**
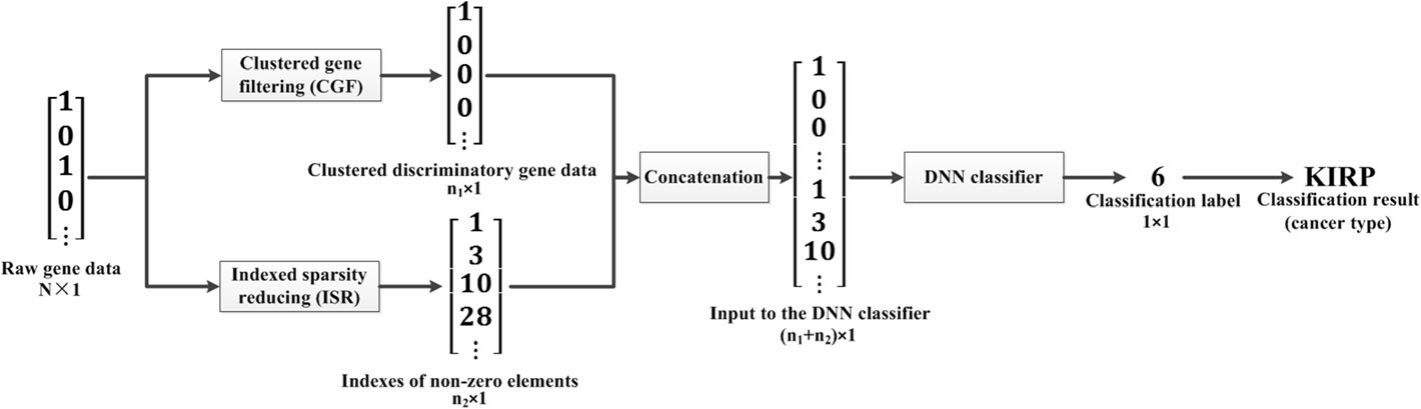
Flowchart of the proposed DeepGene method. The raw gene data is first pre-processed by the clustered gene filtering (CGF) and the indexed sparsity reduction (ISR), respectively, and then fed into the DNN classifier. The output label from the DNN indicates the cancer type of the input gene sample

The flowchart of DeepGene is shown in Fig. 1. We conduct experiments on the proposed TCGA-DeepGene dataset, and DeepGene is evaluated against three widely adopted classification methods for SMCC. The results demonstrate that DeepGene has generated significantly higher performance in terms of testing accuracy against the comparison methods.

## Methods

DeepGene has three major steps, namely clustered gene filtering (CGF), indexed sparsity reduction (ISR), and DNN-based classification. The CGF and ISR are two independent pre-processing modules, the results of which are then concatenated in the final DNN classifier.

### Clustered gene filtering

The CGF step is based on the mutation occurrence frequency of the gene data, and its workflow is summarized in Table 1. Let *A* ∈ {0, 1}^*m* × *n*^ be the matrix of raw data with binary value, where the *n* columns correspond to the *n* samples (cases) in the dataset, and the *m* rows correspond to the *m* genes per sample. The binary value indicates whether a mutation is observed:

**Table 1:** Workflow of Clustered Gene Filtering (CGF)

**Input**: Gene data matrix *A* ∈ {0, 1}^*m* × *n*^, distance threshold *d*_*CGF*_, group element threshold *n*_*CGF*_. 1: Sum *A* by row and sort the result in descent order, and then obtain the sorted index *A*_sum_^*^; 2: Initialize each element as ungrouped; 3: For each ungrouped element *i* in *A*_sum_^*^: (a) For each ungrouped element *j* in *A*_sum_^*^ other than *i*: i. Calculate the similarity *d*(*A*(*i*, :), *A*(*j*, :)); ii. If *d*(*A*(*i*, :), *A*(*j*, :)) > *d*_*CGF*_, assign *j* into the group of *i*; 4: Set the output gene index set *g*_*out*_ = ∅; 5: For each group *c* of *A* after step 3: (a) If group element number *n*_*c*_ ≥ *n*_*CGF*_, select the top *n*_*CGF*_ genes with the highest mutation occurrence frequency as *g*_*c*_; (b) *g*_*out*_ = *g*_*out*_ ∪ *g*_*c*_; 6: Apply the index set *g*_*out*_ on *A* and get the filtered gene data *A*_*CGF*_ = *A*(*g*_*out*_, :); **Output**: *A*_*CGF*_, i.e. the gene data after CGF	

$$ \mathrm{A}\left(i,j\right)=\left\{\begin{array}{c}\hfill 1\hfill \\ {}\hfill 0\hfill \end{array}\right.\begin{array}{c}\hfill \mathrm{if}\kern0.5em \mathrm{mutation}\kern0.5em \mathrm{obsereved}\kern0.5em \mathrm{at}\kern0.5em \mathrm{gene}\kern0.5em \mathrm{i}\kern0.5em \mathrm{of}\kern0.5em \mathrm{sample}\kern0.5em \mathrm{j}\hfill \\ {}\hfill \mathrm{otherwise}\hfill \end{array}. $$

We first sum *A* by row, and concatenate the result with the row indexes for later reference (step 1 in Table 1):

$$ {A}_{sum}=\left[\begin{array}{c}\hfill 1\hfill \\ {}\hfill \vdots \hfill \\ {}\hfill m\hfill \end{array}\kern0.5em  sum\left(A, axis= row\right)\right]. $$

Since the genes with higher occurrence frequency are of more interest, the rows of *A*_*sum*_ are sorted in descending order by the second column as *A*_sum_^*^. After that, we only keep its index column:

$$ {A}_{sum}^{*}={A}_{sum}^{*}\left(:,1\right). $$

The next step is to group *A*_sum_^*^ by inter-gene similarity (step 3 in Table 1). For two 1 × *n* gene samples *p* and *q*, we use the Jaccard coefficient as their inter-sample similarity *d*(*p*, *q*):

$$ d\left(p,q\right)=\frac{sum\left(p\&q\right)}{sum\left(p\Big|q\right)}, $$

where “&” and “|” stand for logical AND and OR.

Starting from *A*_sum_^*^(1), which stands for the index of the gene with the highest occurrence frequency, we calculate its similarity with each of the following genes. If their similarity is larger than a predefined threshold *d*_*CGF*_, the latter gene is merged into the group of *A*_sum_^*^(1). After the loop for *A*_sum_^*^(1), we conduct the loop for the next ungrouped element in *A*_sum_^*^, until all the genes are grouped with a unique group ID.

The final step is to filter the elements from each group and form the discriminatory subset. We do this by selecting the top *n*_*CGF*_ genes in each group with the highest mutation occurrence frequency, where *n*_*CGF*_ is another predefined threshold. Groups that have fewer than *n*_*CGF*_ elements are discarded. All of the selected genes are then united as the result of CGF (steps 5 and 6 in Table 1).

### Indexed sparsity reduction

Although the CGF can effectively locate the discriminatory gene subset and filter out the majority of irrelevant genes, it is still probable that the selected gene subset being highly sparse, i.e. most of the elements in *A*_*CGF*_ are zeros. The high sparsity is likely to obscure any distinguishable feature in the gene data and severely hinder the classification. Hence, an effective process in reducing the gene data sparsity is highly desired.

**Fig. 2:**
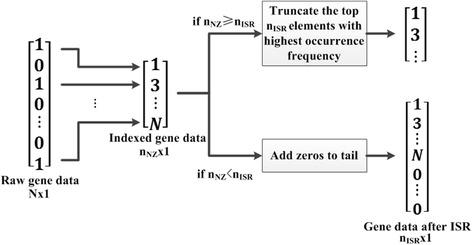
Flowchart of the Indexed Sparsity Reduction (ISR) step. After indexing of the non-zero elements, if *n*_*NZ*_ ≥ *n*_*ISR*_, select the top *n*_*ISR*_ non-zero elements that have the highest occurrence frequency; if *n*_*NZ*_ < *n*_*ISR*_, we conduct zero-padding to the tail of the output data so that it has the length of *n*_*ISR*_

To address the data sparsity issue, we propose the indexed sparsity reduction (ISR) procedure, which minifies the sparsity by converting the gene data into the indexes of its non-zero genes. For a 1 × *n* gene sample *p* ∈ {0, 1}^1 × *n*^, let the number of its non-zero element be *n*_*NZ*_. We set a pre-defined threshold *n*_*ISR*_. If *n*_*NZ*_ ≥ *n*_*ISR*_, find the indexes of its top *n*_*ISR*_ non-zero elements that have the highest occurrence frequency in *A*_sum_^*^ of the previous section, and these *n*_*ISR*_ indexes are listed in ascending order as a vector *p*_*ISR*_, which is the output of ISR; if *n*_*NZ*_ < *n*_*ISR*_, we conduct zero-padding to the tail of *p*_*ISR*_ to make it has the length of *n*_*ISR*_. The workflow of ISR is illustrated in Fig. 2.

The significance of ISR is apparent. For each gene sample *p*, ISR filters out the majority of its zero elements and leaves most (if *n*_*NZ*_ ≥ *n*_*ISR*_) or all (if *n*_*NZ*_ < *n*_*ISR*_) of its non-zero elements. Since *n*_*ISR*_ ≪ *length*(*p*), the percentage of zero elements will drop dramatically after ISR, which means the impact of data sparsity will be significantly suppressed.

### DNN-based classifier

As introduced in the previous two sections, both CGF and ISR have their own advantages when conducted alone. However, the performance can be even higher if they are combined together (see more details in the “Evaluate the effect of combining CGF and ISR” Section). We thus combine both CGF and ISR as the pre-processing for our DNN-based classifier.

As shown in Fig. 1, the raw gene data is processed by CGF and ISR, separately, and then concatenated as the input of the DNN classifier. The concatenation is conducted by appending the output of ISR to the tail of the output of CGF, by which the two outputs form a new and longer data vector. The classifier is a feed-forward artificial neural network with fixed input and output size, and multiple hidden layers for data processing. For a hidden layer *l*, its activation (or output value to the next layer) is computed as:

$$ {x}_l=f\left({z}_{l-1}\right), $$

where *f* is the activation function, *z*_*l*_ is the total weighted sum of the input:

$$ {z}_l={W}_l{x}_l+{b}_l, $$

where *W*_*l*_ and *b*_*l*_ are the weight matrix and bias vector of layer *l* (to be learned in training). In our case, we adopt the ReLU [40] function as *f*, and *x*_1_ is the input gene data after pre-processing. The size of the last layer *L*’s output *x*_*L*_ equals to the number of cancer types *n*_*cancer*_ (*n*_*cancer*_ = 12 in our case). *x*_*L*_ is then processed by a softmax layer [41], and the loss *J* is computed by the logarithm loss function:

$$ J=-{\displaystyle \sum_{i=1}^{n_{cancer}}{y}_i \log {P}_i}, $$

where *y*_*i*_ ∈ {0, 1} is the ground truth label of cancer type *i*, and

$$ {P}_i=\frac{x_L(i)}{{\displaystyle \sum_j \exp \left({x}_L(j)\right)}} $$

is the softmax probability of cancer type *i*.

In training, the loss *J* is transferred from the last layer to the former layers via back-propagation [32], by which the parameters *W* and *b* of each layer are updated. The training then enters the next epoch, and the feed-forwarding and back-propagation are conducted again. The training stops when a pre-defined epoch number is reached. In testing, only the feed-forwarding is conducted (for once) for a testing sample, and the type of cancer *i* corresponding to the largest softmax probability of *P*_*i*_ is adopted as the classification result. The workflow of the DNN classifier is summarized in Table 2, and the complete flowchart of DeepGene is illustrated in Fig. 1.

**Table 2:** Workflow of DNN classifier

**Input**: Gene data matrix *A* ∈ {0, 1}^*m* × *n*^ after CGF and ISR, where rows and columns correspond to samples and genes, respectively; max training epoch *E*_max_. 1: Training: for each training epoch *e* ≤ *E*_max_: (a) For each sample *a*_*i*_ = *A*(*i*, :): i. Conduct feed-forwarding and compute the loss *J*; ii. Conduct back-propagation to update the *W* and *b* 2: Testing: for each sample *a*_*i*_ = *A*(*i*, :): (a) Conduct feed-forwarding and get softmax probability *P*; (b) Adopt the cancer type correspond to max(*P*) as the result of *a*_*i*_. **Output**: Trained network model (training) or classification results for the samples (testing).	

## Results

### Experiment setup

#### Dataset

Our experiments are all conducted on the newly proposed TCGA-DeepGene dataset, which is a re-formulated subset of The Cancer Genome Atlas (TCGA) dataset [39] that is widely applied in genomic researches.

The TCGA-DeepGene subset is formulated by assembling the genes that contain somatic point mutation on each of the 12 selected types of cancer. Detailed sample and point mutation statistics for each cancer type can be found in Table 3. The data is collected from the TCGA database with filter criteria IlluminaGA_DNASeq_Curated updated before April, 2015. The mutation information for a gene is represented by a binary value according to one or more mutation(s) (1) or without mutation (0) on that gene for a specific sample. We assemble a total of 22,834 genes from the 3122 samples, and generate a 22, 834 × 3, 122 binary data matrix (i.e. the original data matrix *A*). This data matrix is the product of our proposed TCGA-DeepGene subset, where each sample (column) is assigned one of the labels {1, 2, …, 12} meaning the 12 types of cancer above.

**Table 3:** Sample and mutation statistics of the TCGA-DeepGene dataset on 12 cancer types

Cancer name	Sample number	Missense mutation	Nonsense mutation	Nonstop mutation	RNA	Silent	Splice_Site	Translation start site	Total mutation
ACC	91	6741	501	15	368	2534	344	42	10,545
BLCA	130	24,067	2142	46	0	9662	528	55	36,500
BRCA	992	55,063	4841	133	3998	17,901	1424	0	83,360
CESC	194	26,606	2716	84	5595	9765	527	0	45,293
HNSC	279	31,416	2545	44	0	12,149	776	0	46,930
KIRP	171	8910	499	17	394	3411	524	0	13,755
LGG	284	5341	378	7	102	2074	294	0	8196
LUAD	230	44,800	3477	46	0	15,594	1377	99	65,393
PAAD	146	21,067	1496	19	859	7936	1005	111	32,493
PRAD	261	9628	563	15	652	3750	513	55	15,176
STAD	288	82,265	4200	92	48	33,344	1868	227	122,044
UCS	56	3070	187	2	234	1114	171	0	4778
Total	3122	318,974	23,545	520	12,250	119,234	9351	589	484,463

To facilitate the 10-fold cross validation in the following experiments, we randomly divide the samples in each of the 12 cancer categories into 10 subgroups, and each time we union one subgroup from each cancer category as the validation set, while all the other subgroups are combined as the training set. This formulates 10 training/validation configurations with fair distributions of the 12 types of cancer, and will be used for the 10-fold cross validation in our following experiments.

#### Constant parameters

For the proposed DNN classifier, the output size is set to 12 (i.e. the 12 types of cancer to be classified); the total training epoch *E*_max_ is set to 50; the learning rate is set to 50-point logarithm space between 10^− 1^ and 10^− 4^; the weight decay is set to 0.0005; and the training batch size (i.e. the number of samples per training batch) is set to 256.

Additionally, in order to facilitate the evaluation of variable parameters, we set each parameter a default value: the distance threshold is set to 0.7; the group element threshold *n*_*CGF*_ is set to 5; the non-zero element threshold *n*_*ISR*_ is set to 800; the hidden layer number and parameters per layer of the DNN classifier are set to 4 and 8192, respectively.

#### Evaluation metrics

For all the evaluations in our experiments, we randomly select 90% (2810) samples for training, and the rest 10% (312) samples for testing. In parameter optimization steps for DeepGene, we adopt the 10-fold cross validation accuracy on the training set as the evaluation metric; on the other hand, in the comparison with widely adopted models, we adopt the testing accuracy as the evaluation metric.

#### Implementation

The CGF and ISR steps are implemented by original coding in MATLAB, while the DNN classifier is implemented on the MatConvNet toolbox [42], which is a MATLAB-based convolutional neural network (CNN) toolbox with various extensibilities.

### Evaluation of design options

#### Determination of CGF’s variable

**Fig. 3:**
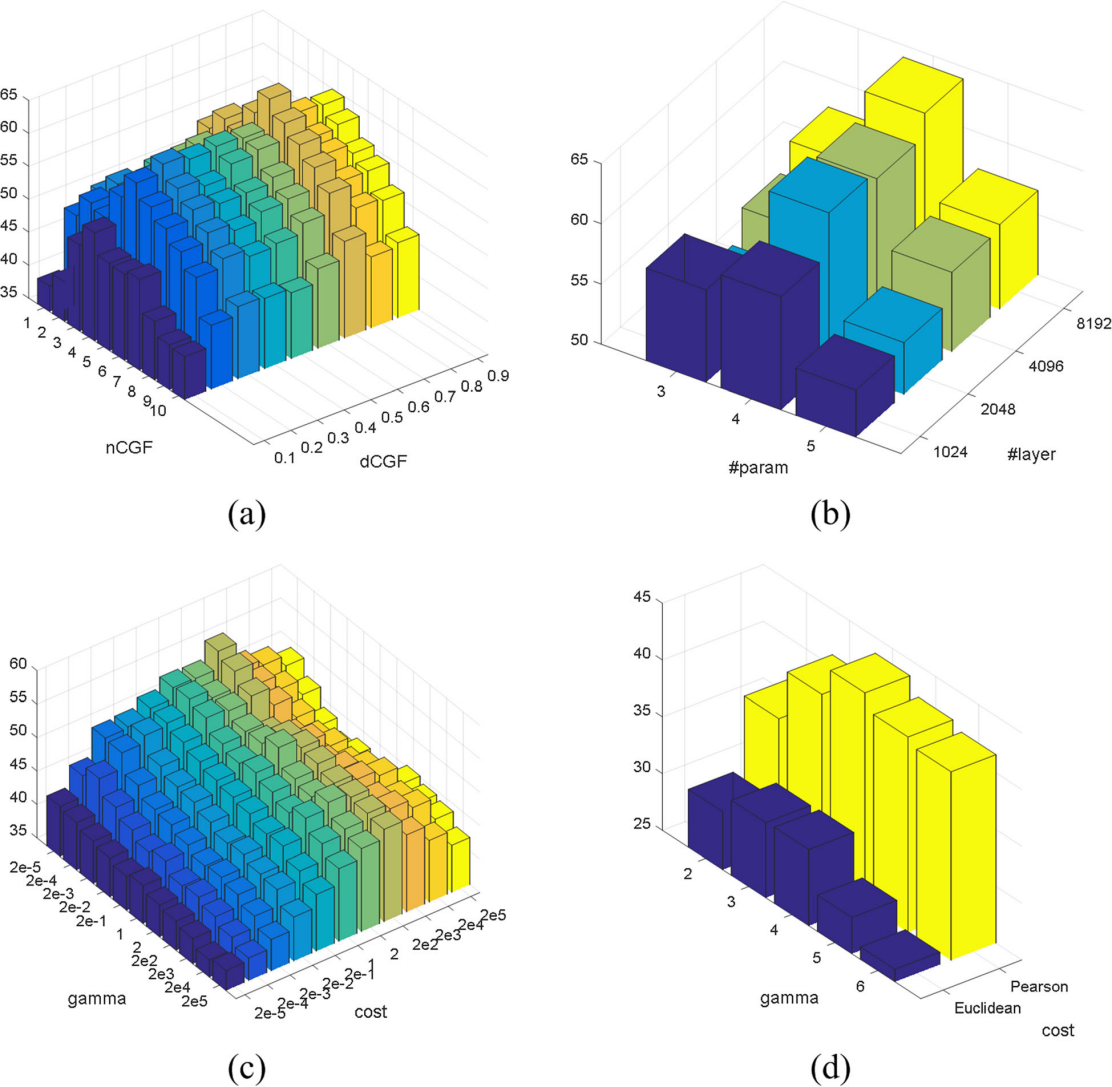
3D bar-plots of parameter estimations for sensitivity review. The Z-axis stands for 10-fold cross validation accuracy. **a** Parameter estimation for *d*_*CGF*_ and *n*_*CGF*_, corresponding to Table *4*; **b** parameter estimation for layer number and parameter number per layer for the DNN classifier, corresponding to Table *5*; **c** parameter estimation for cost and gamma for SVM, corresponding to Table *6*; **d** parameter estimation for Table *7*

There are two variables that need to be experimentally determined for the CGF step, namely the distance threshold *d*_*CGF*_ and the group element threshold *n*_*CGF*_. To determine the two variables, we change them in 2-dimensional manner, while keeping all the other variables the default values as described in the “Constant parameters” Section. The corresponding 10-fold cross validation accuracies are listed in Table 4, and the corresponding 3D bar-plot to present sensitivity is shown in Fig. 3a. We adopt *d*_*CGF*_ = 0.7 and *n*_*CGF*_ = 5 for the following experiments based on the observed experimental results, since they contribute to the optimal performance.

**Table 4:**
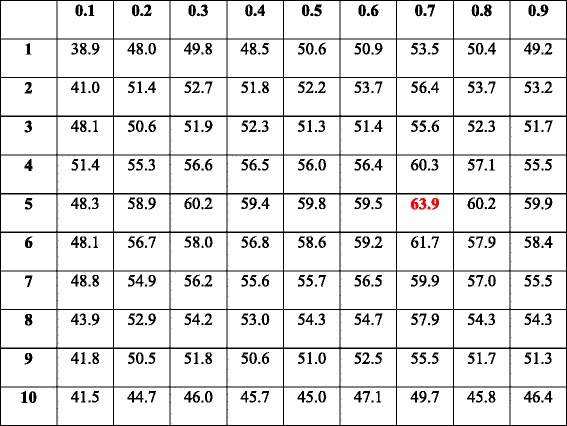
10-fold cross validation accuracies (%) of DeepGene with different *n*_*CGF*_ (row) and *d*_*CGF*_ (column)

The optimal result is marked in red. Mean accuracy: 53.0%; standard deviation: 5.01%; maximum accuracy: 63.9%; minimum accuracy: 38.9%. The corresponding 3D bar-plot is shown in Fig. 3a for sensitivity review

#### Determination of ISR’s variable

**Fig. 4:**
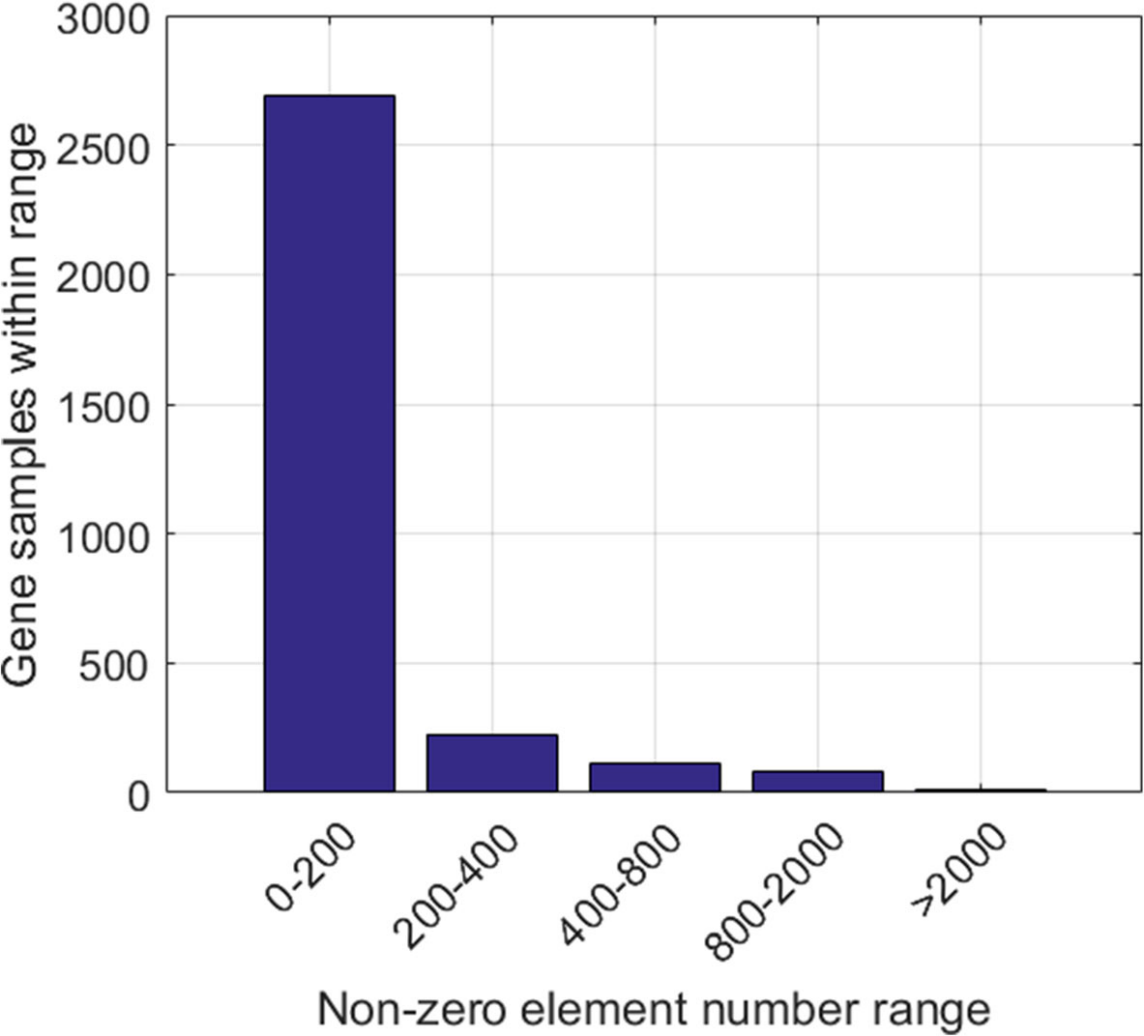
Non-zero element distribution of the gene samples in the TCGA-DeepGene dataset. Ninety-seven percent of all the 3122 samples have no more than 800 non-zero gene elements

The non-zero element threshold *n*_*ISR*_ needs to be experimentally determined for the ISR step. We monitor the number of non-zero elements for each sample in the dataset, and plot the corresponding histogram in Fig. 4. It is seen that 3030 (or more than 97%) of the 3122 samples have less than 800 non-zero genes among the total 22,834 genes. We thus adopt *n*_*ISR*_ = 800, which not only concentrates the data to the non-zero elements, but also greatly shrinks the data length.

#### Determine the network architecture

We also need to determine the network architecture for the DNN classifier, which involves two variables: the hidden layer number (#layer) and the parameter number per layer (#param). Enlightened by [43], we monitor the classifier’s 10-fold cross validation accuracy with various hidden layer numbers and parameter numbers, the results of which are listed in Table 5, and the corresponding 3D bar-plot to present sensitivity is shown in Fig. 3b. We see that the performance reaches optimal at #layer = 4 and #param = 8192. These values are thus adopted in our following experiments.

**Table 5:**
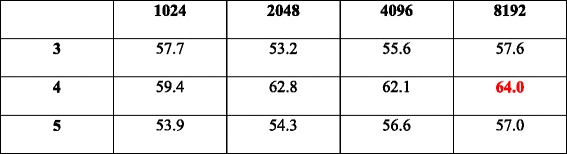
10-fold cross validation accuracies (%) of DeepGene with different #layer (row) and #param (column)

The optimal result is marked in red. Mean accuracy: 57.9%; standard deviation: 3.42%; maximum accuracy: 64.0%; minimum accuracy: 53.2%. The corresponding 3D bar-plot is shown in Fig. 3b for sensitivity review

#### Evaluate the effect of combining CGF and ISR

After determining the related parameters for the three steps of DeepGene, we evaluate the impact of our two major innovations, i.e. CGF and ISR. It is mentionable that we conduct CGF and ISR separately and concatenate their results (as shown in Fig. 1) instead of conducting them consecutively. The reason is that the outputs of CGF and ISR are binary data and index data, respectively. Consecutive conduction will only leave the index data (from ISR), while separate conduction can benefit from both the binary data and the index data, thus introduces less bias.

Based on Fig. 1, we compare the performances of the DNN classifier with different configurations:

1. CGF and ISR (i.e. the proposed input structure);
2. Only CGF (the upper half of Fig. 1);
3. Only ISR (the lower half of Fig. 1);
4. Neither CGF nor ISR (use the raw gene data instead).

**Fig. 5:**
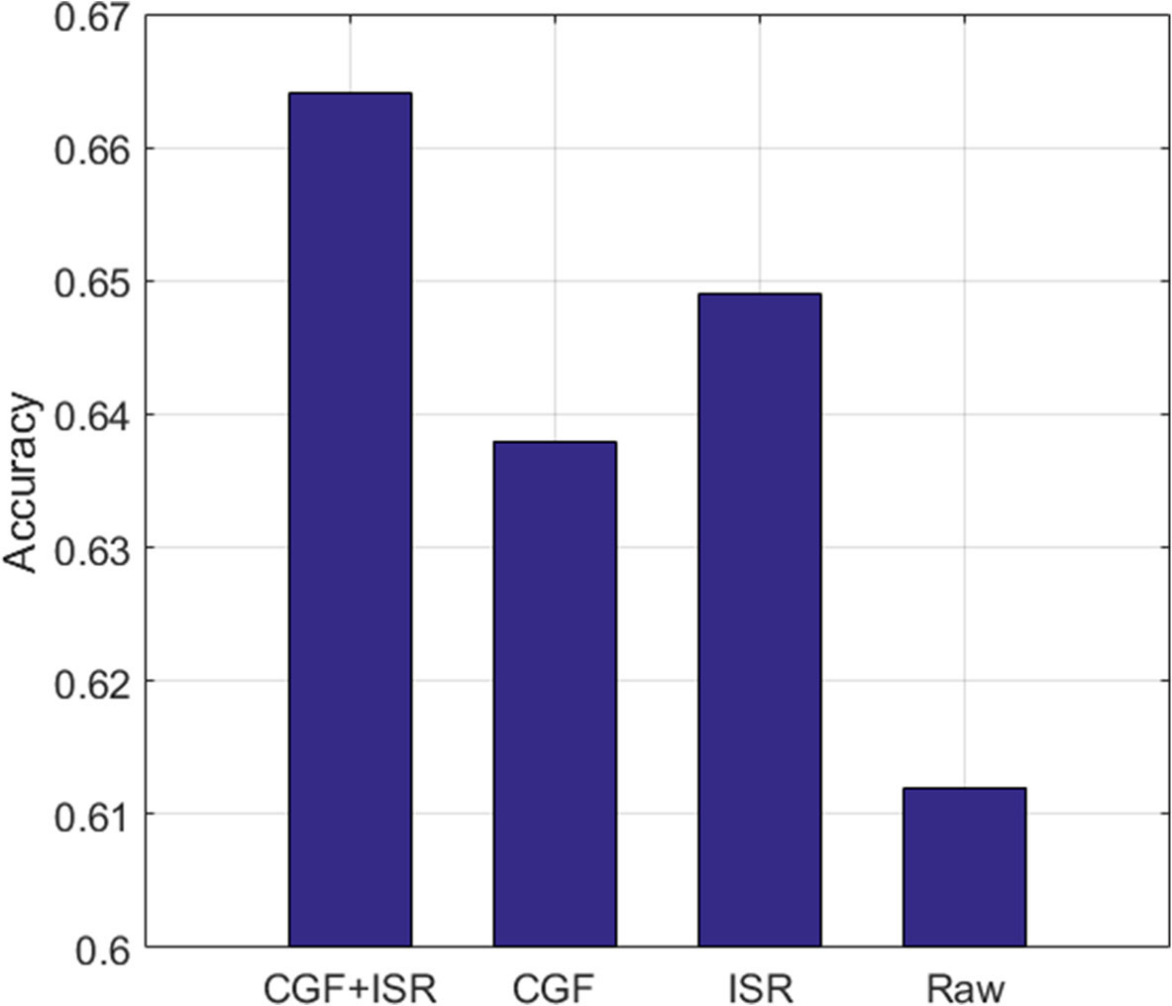
10-fold cross validation accuracy of DeepGene with different design options. Performance comparison of the complete DeepGene input structure (CGF + ISR), CGF only, ISR only and raw gene data. The complete DeepGene shows significant advantage against the other three options

The 10-fold cross validation results are shown in Fig. 5. It is clearly observed that the complete CGF + ISR outperforms both CGF and ISR when conducted alone, and also significantly outperforms the raw data without any pre-processing.

### Comparison with widely adopted models

We then select three most representative data classifiers that are commonly used in SMCC as comparison methods, namely Support Vector Machine (SVM) [28], *k*-Nearest Neighbors (KNN) [44] and Naïve Bayes (NB) [45]. In order to exhibit the pre-processing effect of CGF and ISR, all the comparison methods use raw gene data as inputs. The three methods are set up as below.

**SVM:** we use the LIBSVM toolbox [46] in implementing the SVM. Based on the results of a previous work for gene classification [26], the kernel type (−t) is set to 0 (linear kernel). Note that due to the feature set is high dimensional, the linear kernel is suggested over the RBF (Gaussian) kernel [46]; this suggestion is consistent to our trial and error experience on this problem. A 10-fold cross validation is conducted to optimize the parameters cost (−c) and gamma (−g), and the other parameters are set as their default values. The cross validation results are shown in Table 6, and the corresponding 3D bar-plot to present sensitivity is shown in Fig. 3c. We adopt 2^2^ = 4 and 2^‐ 5^ = 0.0313 for -c and -g, respectively, which lead to the best results in Table 6.

**Table 6:**
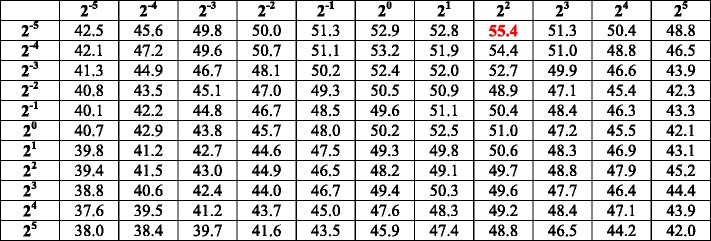
10-fold cross validation accuracy (%) of SVM with different cost (row) and gamma (column) parameters

The optimal result is marked in red. Mean accuracy: 46.6%; standard deviation: 3.97%; maximum accuracy: 55.4%; minimum accuracy: 37.6%. The corresponding 3D bar-plot is shown in Fig. 3c for sensitivity review

**KNN:** we compare the performances of Euclidean distance and Pearson correlation coefficient, which are the two most commonly used similarity measures in gene data analysis [26]. The 10-fold cross validation results of the two similarity measures with different neighborhood numbers are shown in Table 7, and the corresponding 3D bar-plot to present sensitivity is shown in Fig. 3d. We adopt the Pearson correlation coefficient and set the neighborhood number to 4, which lead to the optimal validation accuracy.

**Table 7:**
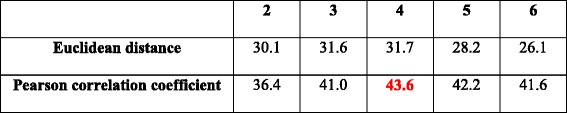
10-fold cross validation accuracies (%) of KNN with different similarity measures (row) and neighborhood numbers (column)

The optimal result is marked in red. Mean accuracy: 35.3%; standard deviation: 5.63%; maximum accuracy: 43.6%; minimum accuracy: 28.2%. The corresponding 3D bar-plot is shown in Fig. 3d for sensitivity review

**NB:** following [47], the average percentage of non-zero elements in the samples of each cancer category is set as the prior probability.

**Fig. 6:**
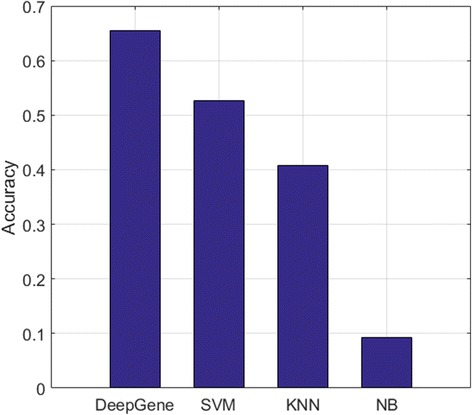
Testing accuracy of DeepGene against three widely adopted classifiers. DeepGene is clearly advantageous to the comparison methods

**Fig. 7:**
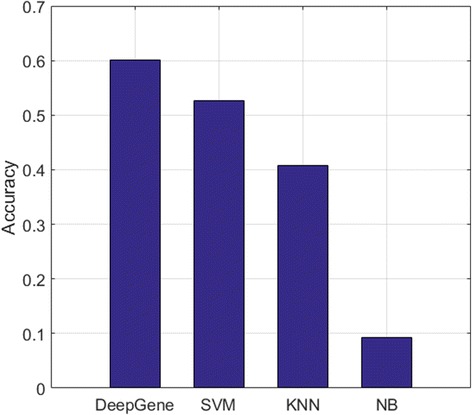
Testing accuracy of DeepGene against three widely adopted classifiers with raw gene input data. All methods use raw gene data as input. The DNN classifier is still favorable against the other methods

In the performance comparison between different models, the testing accuracy is adopted as the evaluation metric (see the “Evaluation metrics” Section), which is generally slightly lower than the 10-fold validation accuracy of the corresponding model. The experiment results are plotted in Fig. 6. DeepGene shows significant advantage against all the three comparison methods. The performance improvements are 24.3% (65.5% vs. 52.7%), 60.5% (65.5% vs. 40.8%) and 710% (65.5% vs. 9.23%) against SVM, KNN and NB, respectively. To further validate the performance of the DNN classifier itself without CGF and ISR, we also record the accuracy of the DNN classifier with raw gene data, which is the same input as the comparison methods. The results are shown in Fig. 7, in which the DNN classifier still has the optimal accuracy (60.1%) against all of the comparison methods.

## Discussion

### Clustered gene filtering

The main purpose of the CGF step is to filter out irrelevant genes in the samples and locate the candidate discriminatory gene subset. It first groups the genes based on popularity (mutation occurrence frequency) and inter-sample similarity, and then selects the top genes in each group, and finally unites all the genes selected as the output.

The two required parameters, *d*_*CGF*_ and *n*_*CGF*_, are experimentally determined (as shown in Table 4). The two adopted values, *d*_*CGF*_ = 0.7 and *n*_*CGF*_ = 5, are in the midstream of the evaluation ranges, which are more reliable than the marginal values.

By comparing the performances of the CGF against raw gene data, as the second and fourth bars in Fig. 5 indicate, the CGF has exhibited significant performance boosting. It raises the validation accuracy by 4.25% (from 61.2 to 63.8%), and also contributes to the high performance of the combined CGF + ISR input structure. The advantage of CGF lies in its ability to mask out the majority of irrelevant genes, thus maximally suppress their negative influence, and only focus the data to the discriminatory gene subset.

### Indexed sparsity reduction

The ISR step is meant to reduce the data sparsity by converting the gene data into the indexes of its non-zero elements. In that case only the non-zero elements’ information is left, while all the zero elements are discarded. The data sparsity will thus be tremendously reduced, making the subsequent classifier only focus on the informative non-zero elements.

The required parameter *n*_*ISR*_ is experimentally determined. We monitor the non-zero element distribution among all of the 3122 samples in the TCGA-DeepGene dataset, and record the non-zero element range of each sample. Figure 4 indicates that 97% of the samples have no more than 800 non-zero elements (which are only 3.5% of the total 22,834 genes per sample). We thus set *n*_*ISR*_ = 800, which is able to reduce the majority of the data sparsity while maximally reserving the discriminatory information of the samples.

Like CGF, ISR has exhibited significant contribution in improving the performance of our classifier, as the third bar in Fig. 5 indicates. It raises the accuracy against raw gene data by 6.05% (from 61.2 to 64.9%), which is even more significant than what the CGF contributes. We attribute ISR’s advantage to its remarkable reduction of the gene data sparsity. It is also mentionable that ISR exhibits more strength when combined with CGF, as the first bar in Fig. 5 indicates. This can be explained by the synergy effect of binary gene data and indexed gene data.

Furthermore, we note that ISR conducts lossless conversion when *n*_*NZ*_ ≤ *n*_*ISR*_, i.e. the indexed data can be readily converted back to the original binary data if necessary.

### Data optimization by CGF and ISR

Besides aiding our DeepGene method, the CGF and ISR steps can also benefit other classification methods for input data optimization. To evaluate the optimization effect, we apply CGF + ISR to the three classifiers SVM, KNN and NB discussed in the “Comparison with widely adopted models” Section, and record their testing accuracies before and after the input data optimization. For fair comparison, the parameters of the classifiers remain the same.

**Fig. 8:**
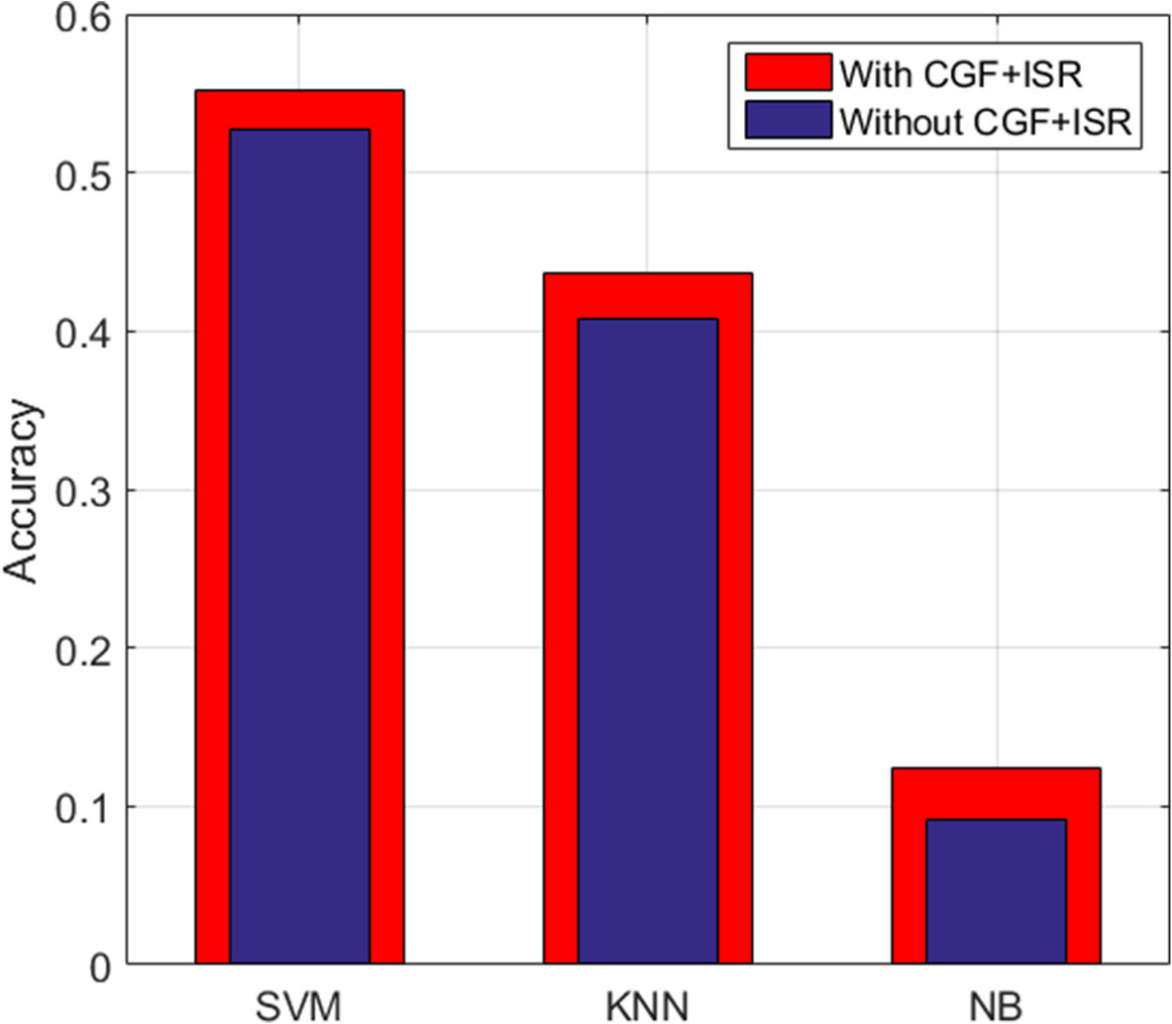
Testing accuracies of three widely adopted classifiers with and without CGF + ISR for input data optimization. Applying CGF + ISR can notably refine the input data, thus improve the testing accuracies of the classifiers

Figure 8 shows the accuracy change before and after the input data optimization of CGF + ISR. It is observed that applying CGF + ISR can notably refine the input data, thus improve the testing accuracies of the classifiers. We also note that by applying CGF + ISR, the accuracy improvements of the three classifiers are not as large as that of DeepGene. Since DeepGene is based on DNN, it is more advantageous in processing complicated data structures, thus can benefit from CGF + ISR more.

### DNN classifier

The DNN classifier is the mainstay of DeepGene, which conducts the classification and generates the final output. Figure 6 has shown the significant advantage of DeepGene against three widely adopted classifiers, among which DeepGene exhibits at least 24% of performance improvement. To examine the performance of the DNN classifier itself without the pre-processing steps of CGF and ISR, we also record the accuracy of the DNN classifier with raw gene data in Fig. 7, which has shown that the DNN classifier still generates the best accuracy (60.1% against the second best 52.7% of SVM).

To further validate that the 10-fold validation accuracy of DNN is indeed higher than that of SVM, we assume that these two classifiers are independent of each other, and conduct *t*-test with the null hypothesis that these two classifiers have equal validation accuracy under the significance of 0.001. The sample standard deviation of DNN and SVM are recorded as ${s}_{X_1}=1.51\%=0.0151$ and ${s}_{X_2}=2.12\%=0.0212$, respectively. The *t* statistic is then calculated as:

$$ t=\frac{{\overline{X}}_1-{\overline{X}}_2}{s_{X_1{X}_2}\cdot \sqrt{\frac{2}{n}}}=\frac{0.601-0.527}{3.387e-4\times \sqrt{\frac{2}{10}}}=488.5, $$

where

$$ {s}_{X_1{X}_2}=\frac{s_{X_1}^2+{s}_{X_2}^2}{2}=3.387e-4. $$

Here, the degree of freedom is *n* − 1 = 9. By checking the one-tailed significance table, the corresponding *t* statistic of the *p*-value 0.001 is 3.922, which is far less than our *t* = 488.5. Hence the null hypothesis is rejected in favor of the alternative hypothesis, and we prove that the 10-fold cross validation accuracy of DNN is indeed higher than that of SVM. It is notable that using the DNN alone is the lowest configuration of DeepGene (see Fig. 5), and SVM has the highest performance out of the three comparison classifiers. As a result, our *t*-test above has also proved that DeepGene is indeed higher in performance against all of the three comparison methods.

We attribute the advantage of the DNN classifier to its capacity in extracting the complex features of the input data. The multiple nonlinear processing layers make the DNN especially suitable in processing complex data that are too tough for conventional linear classifiers such as linear kernel SVM. We also note that DeepGene is just one of our initial trials for DNN-based gene data processing, but has already shown promising results against widely adopted methods. The DNN classifier has the potential to show greater advantages towards more complex (e.g. images or multi-dimensional gene data) and large-scale data to conventional classifiers, which will be discussed in our future works.

### Limitation and future study

Currently DeepGene is only tested on datasets of somatic point mutations with known cancer types, i.e., the histological biopsy sites are already known. Therefore, in this study, DeepGene only demonstrates the power of capturing complex association between somatic point mutation and cancer types, and more of its application potentials will be evidenced by tumor samples with completely unknown cancer type information (such as CTC or ctDNA data) in our future works. The association between point mutation and other genetic aberrations such as copy number variance, translocation, and small insertion and deletion will also be covered in our future works. It will be proved that to a large extent, adopting point mutation alone is good enough for cancer type or subtype classification.

## Conclusions

In this paper, we propose the DeepGene method for somatic point mutation based cancer type classification. DeepGene consists of three major steps. The CGF step concentrates the gene data with mutation occurrence frequency; the ISR step reduces the gene data sparsity with the indexes of non-zero elements; and in the final step, the DNN-based classifier takes the processed data and generates the classification result with high-level data feature learning.

We conduct experiments on the compiled TCGA-DeepGene dataset, which is a reformulated subset of the TCGA dataset with mutations on 12 types of cancer. Controlled variable experiments indicate that CGF, ISR and DNN classifier all have significant contribution in improving the classification accuracy. We then compare DeepGene with three widely adopted data classifiers, the results of which exhibit the remarkable advantages of DeepGene, which has achieved > 24% of performance improvement in terms of testing accuracy against the comparison classification methods.

We demonstrated the advantages and potentials of the DeepGene model for somatic point mutation based gene data processing, and we suggest that the model can be extended and transferred to other complex genotype-phenotype association studies, which we believe will benefit many related areas. As for future studies, we will refine our model for other complex and large-scale data, as well as broadening our training dataset, so that the classification result can be further improved.
